# Frequency following responses and rate change complexes in cochlear implant users

**DOI:** 10.1016/j.heares.2021.108200

**Published:** 2021-05

**Authors:** Robin Gransier, Franҫois Guérit, Robert P. Carlyon, Jan Wouters

**Affiliations:** a KU Leuven, Department of Neurosciences, ExpORL, Herestraat 49, Box 721, Leuven 3000, Belgium; b Cambridge Hearing Group, MRC Cognition and Brain Sciences Unit, University of Cambridge, 15 Chaucer Road, Cambridge CB2 7EF, United Kingdom

**Keywords:** Cochlear implants, Temporal processing, Pitch, Rate, Frequency following response, Auditory change complex, Stimulation artifacts, Artifact removal

## Abstract

The upper limit of rate-based pitch perception and rate discrimination can differ substantially across cochlear implant (CI) users. One potential reason for this difference is the presence of a biological limitation on temporal encoding in the electrically-stimulated auditory pathway, which can be inherent to the electrical stimulation itself and/or to the degenerative processes associated with hearing loss. Electrophysiological measures, like the electrically-evoked frequency following response (eFFR) and auditory change complex (eACC), could potentially provide valuable insights in the temporal processing limitations at the level of the brainstem and cortex in the electrically-stimulated auditory pathway. Obtaining these neural responses, free from stimulation artifacts, is challenging, especially when the neural response is phase-locked to the stimulation rate, as is the case for the eFFR. In this study we investigated the feasibility of measuring eFFRs, free from stimulation artifacts, to stimulation rates ranging from 94 to 196 pulses per second (pps) and eACCs to pulse rate changes ranging from 36 to 108%, when stimulating in a monopolar configuration. A high-sampling rate EEG system was used to measure the electrophysiological responses in five CI users, and linear interpolation was applied to remove the stimulation artifacts from the EEG. With this approach, we were able to measure eFFRs for pulse rates up to 162 pps and eACCs to the different rate changes. Our results show that it is feasible to measure electrophysiological responses, free from stimulation artifacts, that could potentially be used as neural correlates for rate and pitch processing in CI users.

## Highlights

• Frequency following responses can be obtained free from stimulation artifacts in cochlear implant users.
• Auditory change complexes can be evoked with pulse rate changes in cochlear implant users.
• Electrophysiological responses can potentially be used for the assessment of rate processing in cochlear implant user.

## 1 Introduction

Most contemporary cochlear-implant (CI) speech-processing strategies apply fixed-rate pulse trains to all electrodes, with each pulse train being modulated by the sound's envelope on a restricted frequency region (Wouters et al., 2015). As a result, the fundamental frequency (F0) of periodic sounds is encoded by the rate of amplitude modulation (AM) applied to one or more electrodes (Müller et al., 2012). This form of F0-encoding differs markedly from the predominant pitch mechanism in normal-hearing listeners, which combines temporal and/or place-excitation information across individual low-numbered harmonics, each of which is resolved by the filtering properties of the basilar membrane (Plack et al., 2014). Furthermore, the AM that occurs in CI processing strategies is often quite shallow and not aligned across channels. A number of strategies, aimed at improving F0-encoding, have been proposed, either by explicitly enhancing and/or aligning the modulations (Geurts and Wouters, 2004, 2001; Green et al., 2012; Laneau et al., 2006; Milczynski et al., 2012; Vandali et al., 2000; Vandalo et al., 2017), or by explicitly encoding the fine structure in the low-frequency channels that are conveyed by apical electrodes (Müller et al., 2012; Riss et al., 2014). One potential reason for the limited success of these algorithms is the presence of a biological limitation on temporal encoding in the electrically-stimulated auditory pathway, which can be inherent to the electrical stimulation itself and/or to the degenerative processes associated with hearing loss. CI users are able to effectively encode pitch and discriminate pulse rates typically up to 300 pulses per second (pps) when a single-pulse-per-period pulse train is presented at a single electrode. This upper limit, however, varies substantially across listeners and electrodes (Carlyon et al., 2018, 2008; Cosentino et al., 2016; Stahl et al., 2016). Furthermore, this upper limit also appears to restrict the encoding of inter-aural time differences in bilateral CI users (Ihlefeld et al., 2015). Understanding the neural basis of this upper limit, and temporal coding in general, is of clinical as well as scientific interest. Especially since the across-subject and across-electrode variations in temporal encoding might serve as an indirect marker of neural health throughout the auditory pathway (Gransier et al., 2020b).

Noninvasive electrophysiological measures could potentially be used to assess the rate encoding abilities of the auditory pathway. Potential measures are the frequency following response (FFR) and the auditory change complex (ACC). The FFR is a phase-locked response to the temporal structure of an acoustic tone or pulse train and is normally measured using rates of at least 100 Hz. Although cortical regions phase-lock to these high rates (Coffey et al., 2016), the main generator(s) that contribute to the scalp-recorded FFR are located in the brainstem (Bidelman, 2018). FFR strength has been associated, in the acoustically-stimulated auditory pathway, with speech-perception-in-noise performance (Coffey et al., 2017a) and pitch perception (Krishnan et al., 2004; Swaminathan et al., 2008; Zhang and Gong, 2017). Although the FFR does not reflect the processing of pitch *per se* (Gockel et al., 2011), it could potentially be used to assess the phase-locking ability of the brainstem to F0 in individual CI users. In contrast, the ACC originates purely from the cortical regions of the auditory pathway (Näätänen and Picton, 1987). The ACC is a classical transient-cortical response, originating from the auditory cortex, which can be elicited by a change in stimulus parameters. In the pitch domain, ACCs can be evoked by frequency changes in normal-hearing humans (Dimitrijevic et al., 2008) and animals (Presacco and Middlebrooks, 2018), and by differences in stimulation electrodes in CI users (Brown et al., 2008; He et al., 2014; Mathew et al., 2018; Mathew et al., 2017; Richardson et al., 2020),

Obtaining electrophysiological responses in CI users, noninvasively by means of EEG, is extremely challenging due to the electrical stimulation artifacts that corrupt the EEG recording. This is especially the case when the neural response is phase-locked to the temporal structure of the stimulus, as occurs for the electrically-evoked FFR (eFFR). Several artifact removal methods have shown to be effective for measuring transient cortical responses such as the electrically-evoked ACC (eACC) (Mathew et al., 2018; Mathew et al., 2017; Mc Laughlin et al., 2013; Viola et al., 2012). However, for phase-locked responses to the envelope or the pulse rate of a stimulus, linear interpolation between consecutive stimulation pulses or parts of the signal is currently the only consistently successful artifact-removal technique for obtaining artifact-free sustained phase-locked responses to electrical stimulation (Bahmer et al., 2018; Deprez et al., 2017b; Gransier et al., 2020b, 2020a, 2016; Hofmann and Wouters, 2012, 2010; Somers et al., 2019). Linear interpolation, however, has only been applied to measure the electrically-evoked auditory steady-state response (eASSR) to the temporal envelope of amplitude-modulated pulse trains (Bahmer et al., 2018; Gransier et al., 2020a, 2016; Hofmann and Wouters, 2012) or to low-rate (< 50 pps) pulse trains (Hofmann and Wouters, 2010). Furthermore, it can only be applied effectively when the duration of the stimulation artifact is shorter than the inter-pulse interval of the stimulus. Artifact removal, based on linear interpolation, for measuring eASSRs to AM pulse trains has been shown to be successful for rates up to 900 pps when stimulating in monopolar mode (Gransier et al., 2020a). These pulse rates are, however, based on the removal of the artifact component at the modulation frequency, which is an order of magnitude lower than that of the carrier pulse rate (Gransier et al., 2020b). The artifact component at the carrier frequency is, however, of importance when measuring eFFRs to single-pulse-per-period pulse trains. Therefore, the applicability of linear interpolation as an artifact-removal method for measuring eFFRs cannot be easily derived from eASSR results evoked with modulated pulse trains. So, in order to use electrophysiological measures to assess the neural mechanism of rate processing and rate-based pitch processing in CI users, it is essential to assess if these responses can be obtained free from stimulation artifacts.

## 2 Rationale

In this technical note we explore whether electrophysiological measures can be obtained from CI users to investigate rate/pitch-processing ability noninvasively and objectively at the level of the brainstem and auditory cortex. Electrophysiological measures in CI users are, however, affected by electrical stimulation artifacts inherent to electrical stimulation. Applying artifact removal and distinguishing between an artificial response and a true neural response is challenging, especially when the stimulation frequency is the same as the response frequency, as is the case for the eFFR. This is especially troublesome when using clinically relevant stimulation configurations, such as stimulating in monopolar stimulation, due to the large stimulation artifacts associated with it (Deprez et al., 2017b; Hughes et al., 2004). Here we assess the effectiveness of linear interpolation to remove the stimulation artifacts from an EEG recording and explore whether eFFRs and eACCs can be obtained, free from stimulation artifacts, to single-pulse-per-period pulse trains and to pulse rate changes between rates from 94 to 196 pps, respectively. Both the eFFR and eACC were evaluated to show the feasibility of assessing rate processing at both the subcortical and cortical level.

## 3 Description of methods

### 3.1 Participant information

Five adult CI users took part (mean age = 50.4, range = 24–69, 4 female). All had a history of long-term hearing impairment and CI use ranged from 0.7 to 22.6 years (see **Table I** for the information about the participants). The study was conducted at ExpORL, KU Leuven, Belgium (4 participants) and Cambridge, U.K. (1 participant, S1), and all participants had a device from Cochlear Ltd. This study was approved by the Medical Ethics Committee of the University Hospital in Leuven (approval number: B32201941114) and the National Research Ethics Committee for the East of England (ref. number 00/327). All methods were carried out in accordance with the relevant guidelines and regulations and written informed consent was obtained from all participants before testing.

### 3.2 Stimulation

To explore whether the eFFR and eACC can be used as an objective measure of rate processing in CI users we assessed the phase-locking ability of the brainstem by means of the eFFR to four different pulse rates, namely 94, 128, 164, 196 pps. Furthermore, the ability of the auditory cortex to detect changes in rate was assessed with the eACC to changes from 94 pps (base rate) to 128, 164, and 196 pps (deviant rates). We assessed both the eFFR and eACC in a single experiment, by constructing a stimulation sequence (i.e. a single trial) that consisted out of a base rate (94 pps) and a deviant rate that was either 128, 164, or 196 pps. Each part of a trial had a duration of 2.048 s, and pulse rates were adjusted so that an integer number of pulse rates fitted in a single part of a trial (only rounded numbers are reported). Pulse rates below 200 pps were chosen as they are well within the range of rates that CI users perceive (Zeng, 2002). In addition, the changes in rate, which were 36, 72, and 108% were multiples of the ~35% rate change that most CI users are able to detect (Carlyon et al., 2008). Furthermore, these approximately-equally-spaced pulse rates enable a correct distinction between a neural response and a stimulation artifact. One efficient way to determine correct artifact removal and the presence of a phase-locked neural response is to assess the phase lag of a response at different pulse rates (Gransier et al., 2016; Hofmann and Wouters, 2012). The obtained group delay will correspond to the latency of the generator if the eFFR across pulse rates originates from the same generator(s) (i.e. there is a linear relationship between the pulse rates and the response phase across pulse rates). In contrast, an artifact-dominated response will yield a group delay of 0 ms (Gransier et al., 2016; Hofmann and Wouters, 2012). In order to estimate the group delay accurately, two criteria need to be met: first the responses need to be significant and second the difference in phase lag between two neighboring rates used for testing should not be more than 360°. If the latter requirement is not met, then erroneous unwrapping will occur. Based on the literature on FFRs obtained from normal-hearing listeners we expect FFR latencies for this range of pulse rates between 5 and 10 ms (King et al., 2016), which corresponds, respectively to a phase difference of 180 and 360° over a range of 100 pps. Therefore, the four pulse rates that are used here are sufficient for a correct estimation of the latency of a brainstem source.

All stimuli were generated in Matlab (version R2016b) and were delivered directly to the implant by means of a research interface, which consisted of a laptop with custom-written software interfacing with the Nucleus Implant Communicator (version 3) and connected to the implant through a programming device and an L34 research processor. The hardware and the Nucleus Implant Communicator were provided by Cochlear Ltd. The pulse trains consisted of symmetric biphasic cathodic-first pulses, with a phase width of 25 µs and an interphase gap of 8 µs. The most apical electrode (e22) was used for stimulation in monopolar mode (i.e. both the extracochlear electrode on the casing and the extracochlear ball electrode were used as the return electrode). The most apical electrode was used since this electrode was assumed to have the highest chance of activating neurons of the auditory nerve that innervate apical cochlear regions and that are important for rate encoding (Macherey et al., 2011; Middlebrooks and Snyder, 2010; Stahl et al., 2016).

Stimulation levels were determined behaviorally at the beginning of each experiment by using a 7-point categorical loudness scale (i.e. “inaudible”, “very soft”, “comfortably loud”, “loud”, “very loud”, and “unbearable”). First, the maximum comfort level for the base rate (i.e. 94 pps) was assessed by starting at 10 current levels^1^ and the current was then gradually increased to obtain the most comfortable loudness level which is defined as the current level prior to the transition between “comfortably loud” and “loud” on the categorical loudness scale. The deviant rates (i.e. 128, 164, and 196 pps) were then loudness-balanced to the base rate. This was done to minimize the effect of overall loudness differences between the base and deviant rate on the ACC. During the loudness-balancing task, a two-down, one-up procedure was used and the participant had to indicate which presentation (either the base-rate or the deviant-rate pulse train) was louder, using a two-alternative forced choice paradigm. Step sizes of two or four levels were used depending on the discrimination ability of the participant. The loudness-balancing procedure was stopped after eight reversals and the mean of the last six reversals was used as the loudness-balanced level. Differences between the base rate and the deviant rate were on average 0.7 CU and ranged from 0 to 3 CU with the largest difference always for the largest difference in pulse rates.

**Fig. 1:**
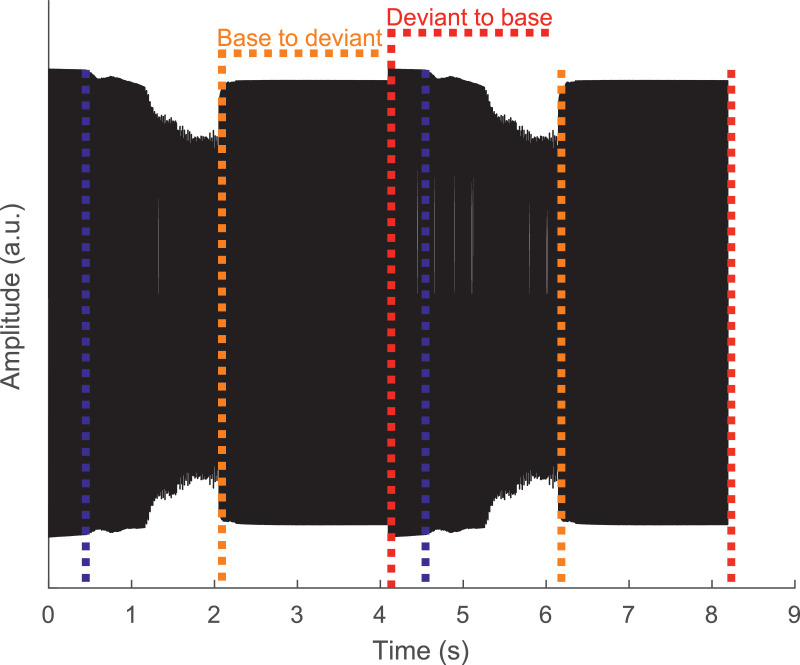
The EEG of two trials of the 94 – 196 pps condition. The first 2.048 s were stimulated at 94 pps and the second 2.048 were stimulated at 196 pps. The red dashed line indicates the deviant-to-base change, the orange dashed line the base-to-deviant change, and the blue dashed line indicates the timepoint where the reduction in amplitude due the compliance issue starts . (For interpretation of the references to color in this figure legend, the reader is referred to the web version of this article.)

For the CIC4 implants used by some participants an additional RF-power pulse was placed before each stimulation pulse. It had the same duration as the stimulation pulse, and the inter-pulse interval between the RF-power pulse and the stimulation pulse was 8 µs. This was necessitated by the greater power requirements of the CIC4 implants, compared to the other implants used by the participants (Table 1). Unfortunately, we found during our experiments that this RF-power pulse was insufficient for Subject S1 and S5 when stimulating at the 94-pps pulse rate (see Fig. 1), leading to a compliance issue that caused the pulse amplitude (and therefore the loudness) to decrease towards the end of each 94-pps 2.048-s epoch. When the pulse rate increased at the start of the deviant-rate epoch the “silent” intervals between the pulses decreased, thereby removing the compliance issue and causing the pulse amplitude to increase. This in turn could have led to a perceived loudness cue that could have biased the ACC for the base-to-deviant change. The ACC to the base-to-deviant change was therefore excluded from the analysis for S1 and S5. Although not included here, we assessed, after observing these issues, what the minimum length needed to be for the CIC4 implants to have no compliance issues at the 94-pps pulse rate by means of an implant-in-the-box and an oscilloscope. No compliance issues were observed for a wide range of loads when the RF-power pulse length was 3 times the stimulation pulse duration.

**Table 1:** Information about the participants.

Subject	Implant	Age (Years)	Implant use (Years)	Implanted ear	Etiology
S1	CI522	43	2.5	Left	Hereditary
S2	CI24RE	69	7.5	Right	Unknown
S3	CI522	67	0.7	Right	Unknown
S4	CI24M	24	22.6	Left	Meningitis
S5	CI422	49	5.8	Right	Unknown

### 3.3 Electrophysiological measures

We used EEG to record the eFFRs and eACCs. A single trial consisted of a 2.024-s base-rate pulse train immediately followed by a 2.024-s deviant-rate pulse train. The stimulation levels were set to those determined during the behavioral assessment of the stimulation levels. 100 trials of a specific combination were concatenated and presented in one block, without containing any silence between trials, and a trigger was sent at the start of each trial from the programming device to synchronize the stimulation and recording. A minimum of 300 trials per condition was presented to each subject for each condition, with the exact number depending on availability. The number of trials actually analyzed (after artifact rejection) is shown for each subject and condition in **Supp. Table I**.

EEG was recorded with an 8-channel Biosemi ActiveTwo Hyper-Rate EEG recording system which was specially designed –to our specifications– to limit the distortion of the artifact waveform and therefore potentially shorten the stimulation artifact duration. We hypothesize that this system allows the removal of the stimulation artifacts and enables one to obtain eFFRs free from stimulation artifacts in CI users when stimulating in monopolar mode. The hyper-rate EEG system is based on the Biosemi ActiveTwo EEG-recording system but with a sample rate of 262.144 kHz/channel and with a built-in analog third-order antialiasing filter having a −3 dB point at 50 kHz. The advantage of this system over other EEG systems is that it has both a high dynamic range and a high sampling rate, hence a high bandwidth (Gransier et al., 2020a), which therefore reduces the distortion and reduces the duration of the artifact waveform (Lio et al., 2018). We used Ag/AgCl active recording electrodes to record the EEG. The recording electrodes were placed at the subject's head using an 6-channel cap according a 10–20 system (Jasper, 1957) layout. Six electrodes were placed on the Fz, Fpz, Cz, P9, P10, Iz locations, and two further electrodes were placed on the left (MaL) and right mastoid (MaR). The DRL and CMS electrodes were placed near the central parietal-occipital location of POz. P10 could not be placed in S3 and S5 due to the location of the CI. Offline referencing was used, and the electrodes placed at Fz, Fpz, Cz, and MaR were used as a reference. Electrode combinations with reference electrodes: Fz, Fpz, and Cz are referred to as vertical montage combinations, whereas the electrode combinations (with the exclusion of Fz, Fpz, and Cz) with MaR as the reference electrode are referred to as the horizontal montage. These different reference positions were included for assessing the artifact removal, since artifact topography can vary across CI users (Gransier et al., 2016).

All EEG recordings, at both sites, were obtained in an electrically shielded (Faraday cage) sound booth, where participants sat in a comfortable chair. To minimize tension in the muscles supporting the head, the head and neck were supported by the chair and occasionally by a cushion. To reduce artifacts caused by movements, participants were asked to move as little as possible during the recording. A silent movie with subtitles was played to ensure that the attentional state was similar in all conditions and for all participants.

### 3.4 Analysis

All off-line signal processing was performed in Matlab (version R2016b). Statistical analyses were performed in R (version 3.5.3). A significance level of 5% was used.

#### 3.4.1 Evaluation of artifact removal

**Fig. 2:**
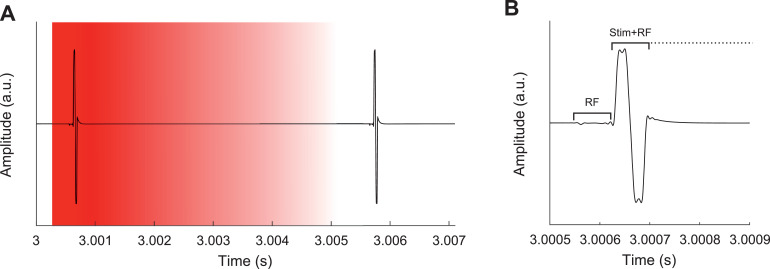
Example of a stimulation artifact recorded ipsilateral to the CI of S1. **A**) illustrates the blanking procedure, where the red section is removed by linear interpolation from the EEG recording after each stimulation pulse. **B**) the first pulse of A showing that the hyper-rate EEG system was able to sample the artifact well. It shows the RF-power pulse, implemented for CIC4 implants followed by the stimulation pulse. The signal shown here is averaged across trials (*n* = 387), unreferenced (i.e. relative to CMS) and filtered to compensate for the drift in the recording over time. (For interpretation of the references to color in this figure legend, the reader is referred to the web version of this article.)

CI-stimulation artifacts were first removed from the raw EEG signal by means of the blanking method; linear interpolation between a prestimulation pulse sample and a poststimulation pulse sample. The prestimulation pulse sample was set at 100 µs before the stimulation pulse for CIC3 implants and 100 µs before the RF-power pulse CIC4 implants. The poststimulation pulse sample was increased in steps of 500 µs to assess the effectiveness of the interpolation length on the removal of the stimulation artifact. This procedure was done as artifact waveforms are known to differ across subjects (Deprez et al., 2017b). After linear interpolation, the time signal of each recording electrode was high-pass filtered using a 2nd order Butterworth high-pass filter with a cutoff frequency of 2 Hz, to remove any drift in the recordings (Fig. 2).

#### 3.4.2 eFFR analysis

We divided each time signal of each recording into individual epochs and sorted those that correspond to the base rate and the deviant rate. The sorted epochs of each recording block were combined. Five percent of the total number of epochs with the highest peak-to-peak amplitude were removed from the recordings to minimize the effect of recording epochs that contained physiological and non-physiological artifacts, such as muscle tension. A Fast Fourier Transform was used to calculate the complex frequency spectrum of each epoch, resulting in a frequency resolution of 0.49 Hz. Referencing was done by subtracting the complex frequency spectrum of the reference electrode from the complex frequency spectrum of each channel and the recording electrode. To compensate for the filter effects on the magnitude of the response, the inverse gain of the high-pass filter was applied to the frequency spectrum of each epoch. This effect was, however, was neglectable for the frequency bins of interest. For each epoch, the response power, amplitudes, and phases were obtained from the complex frequency spectrum corresponding to the pulse rates used during the experiment (i.e. the response spectrum). The mean response amplitude and phase were computed by vector-averaging the complex response spectrum across epochs. The neural background noise was calculated as the standard deviation over epochs divided by the square root of the number of epochs (Gransier et al., 2017). The Hotelling T^2^ (Hotelling, 1931) was used, for each channel, to determine whether the synchronized activity (i.e. the measured response) differed significantly from the non-synchronized neural background activity (Hofmann and Wouters, 2012). This test compares the average real and imaginary components of the response spectrum with the variability across epochs of the response spectrum. We derived the group delay (i.e. the latency of the generator) from the phase slope across the different pulse rates (Picton et al., 2003).

#### 3.4.3 ACC analysis

For the ACC analysis, we referenced the raw-EEG time signals to Cz after linear interpolation and divided each referenced time signal in a base-to-deviant change trial or a deviant-to-base change trial. Trials of the different recording blocks were combined, resulting in an average total of 400 trials per pulse rate (see **Supp. table I**). Five percent of the total number of trials with the highest peak-to-peak amplitude were removed from the recordings to minimize the effect of recording trials that contained physiological and non-physiological artifacts. A low-pass filter (eegfilt, from EEGLAB) with a cutoff frequency of 20 Hz was then applied to derive the ACC waveforms. The Golding et al. (2009) implementation of the Hotelling T^2^ test was used to determine whether an ACC was present. Nine time bins from 50 to 500 ms post stimulus change were used in the Hotelling T^2^ test, each bin containing the average activity within a 50 ms window [for more details see Golding et al. (2009)]. Peaks and latencies were determined using a semiautomatic procedure. Amplitudes were averaged over a 2-ms interval centered on the latency of the peak. The neural background noise level was estimated based on the variance of the 2-ms window mean amplitude across trials and divided by the square root of the number of trials.

## 4 Results

### 4.1 Artifact removal evaluation

The effect of linear interpolation on the removal of the stimulation artifact was assessed on the FFR data. More specifically, we used the response amplitude, the phase, and the derived latency to determine the effect of the blanking length on the response characteristics. Latencies close to zero are considered to be responses dominated by the stimulation artifact, which has a group delay of 0 ms, and latencies between 5 and 10 ms were considered as neural-dominated responses predominately originating from the brainstem regions of the auditory pathway (Bidelman, 2015; King et al., 2016). Effective artifact removal was considered to have occurred when the response amplitude and latency stabilized at a specific blanking length, or when no significant responses could be determined (i.e. neither a neural response or an artifact is present).

**Fig. 3:**
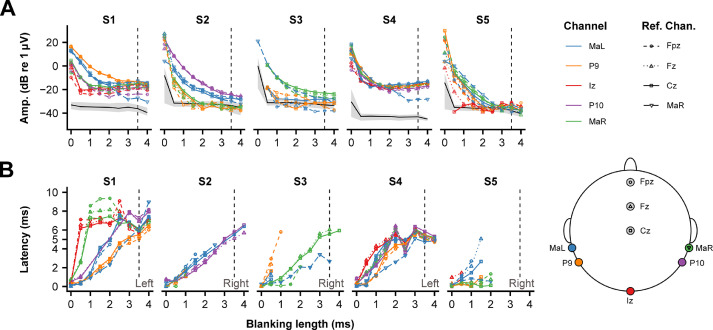
Artifact removal assessment showing **A)** the response amplitude at the stimulation rate (here we used the 164 pps pulse rate) as a function of the blanking length for each individual subject. Colors show the different non-inverting channels and the different symbols and line types show the different reference channels (Ref. Chan.). The solid black line and the shaded area shows the average, and range of noise levels across all recording-electrode configurations as a function the blanking length, respectively. Response amplitudes that were lower than the noise level for a specific combination (i.e. recording-electrode configuration and blanking length) were set to the noise level of that specific combination for illustrative purposes. The dashed vertical line shows the blanking length used in the analysis of the eFFR. **B)** The latency (group delay) of the response as a function of blanking length. The latency was based on the significant responses to the 128- and 164- pulse-rate stimuli. Latencies could only be estimated when both responses were significant. The lack of latencies in e.g. S5 for blanking lengths > 2 ms is a result of this and indicate that the response was not significantly different from the noise level, which is also clear from **Panel A**. The ear of stimulation is shown for each subject in the lower-right corner of subject-specific graph and the EEG-electrode on the scalp is shown on the right-hand side of **Panel B.**

**Fig. 4:**
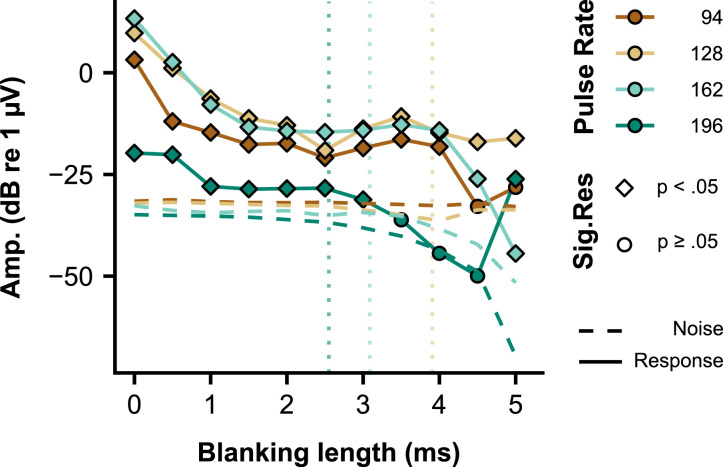
Example of the effect of the blanking length on the response amplitude and the neural background noise amplitude for the different eFFRs to the different pulse rates. Data shown are from a vertical configuration (i.e. left mastoid – Cz) of S1. Vertical dashed lines are shown for illustrative purposes and show the point at which the blanking length exceeds the 50% point of the inter-pulse interval for each specific pulse rate. For illustrative purposes the blanking length was set to a maximum of 5 ms, which corresponds to a removal of 98% of the waveform for the 196-pps pulse rate.

Fig. 3 shows the response amplitude and latency as a function of blanking length. We calculated the latency based on the response phase to the 128- and 164-pps pulse rates. This was done as we found that the 94-pps eFFR phase was not consistent with the latency of a purely brainstem generator (*see* 4**.2 Frequency following responses**), and because 94-pps scalp recordings may have contributions from higher regions of the auditory pathway (Coffey et al., 2017b; Holmes et al., 2018; King et al., 2016; Tichko and Skoe, 2017). Note, however that for every subject, the response amplitude with the 94-pps pulse train showed the same pattern as for the 164-pps pulse train, indicating proper artifact removal (**Supp. Fig. 1A**). Furthermore, the eFFR to the 196-pps pulse train did not yield in any significant responses after blanking at the longer blanking lengths in all subjects (**Supp. Fig 1D**). This is potentially an effect of the blanking operation itself as it removes much of the response waveform, as is evident from the decline in the response amplitude and background noise level at the longer blanking lengths for the higher pulse rates (Fig. 4, **Supp. Fig. 1D**). Effective artifact removal was considered when the amplitude and latency stabilized for the 128- and 164-pps eFFR, and was here determined at 3.5 ms (vertical dash line in Fig. 3 and **Supp.** Fig. 1). Fig. 4 illustrates that this blanking length did not affect the response amplitude for the pulse rates ≤ 164 pps. However, it did for the 196-pps eFFR. Considering that, within a subject, the artifact duration is similar for the same pulse rates, the percentage of EEG response removed from the waveform for a fixed blanking length increases with increasing pulse rate, and was 32.9, 44.8, 56.7, and 67.9% for 94, 128, 164, and 196 pps, respectively. It is therefore expected that the eFFRs to higher pulse rates are more affected by the blanking operation than the eFFRs to the lower pulse rates, since more response waveform is removed when using a specific blanking length. Furthermore, the interaction between the location of the peak of the eFFR waveform and the blanking length can result in the attenuation of the eFFR amplitude.

We used simulations to gain insight in the effect of the blanking procedure on the obtained response amplitudes and noise estimates. First, we simulated the effect of the blanking procedure on single period sinusoids, all with a different phase at the start of the linear interpolation (i.e. a model for a single period FFR). **Supp. Fig. 2 A** shows the effect of the blanking on the waveform for different blanking lengths. It is clear from this illustration that longer blanking lengths result in higher distortions of the waveform. Furthermore, the interpolation differently affects sinusoids with different phases. This is depicted in **Supp. Fig. 2B**, which shows the RMS value of different in phase sinusoids as a function of blanking length. Blanking becomes more problematic when the interpolation durations exceed 60% of the waveform period (**Supp. Fig. 2. B)**. Second, we simulated the effect of the blanking procedure on the neural background noise when a pulse rate of 100 pps would be used. The same blanking procedure was used as in the EEG experiments but then with 300 epochs of 1/f artificially shaped random noise. The same trend in reduction in neural background noise with increasing blanking length was found as in the empirical data when analyzing the frequency bin at the pulse rate (**Supp. Fig. 2C**). These simulations show that blanking lengths that exceed 50% of the inter-pulse interval (i.e. one period of the neural response) make the interpretation of the response difficult due to the distortions that occur both in the response waveform and the neural background noise.

### 4.2 Frequency following responses

Based on the artifact removal analyses, we used the 3.5-ms blanking length to obtain artifact-free eFFRs, and omitted the eFFRs to the 196-pps pulse train, as no reliable response could be obtained when applying the blanking procedure. eFFRs obtained with the vertical montage are reported here, which consisted of the left and right mastoid both referenced to Cz; for subject S2 the right mastoid was replaced by P10 (i.e. in close proximity to the right mastoid) due to a bad electrode contact. The vertical montage was chosen as this resulted in the highest amplitudes and were measurable in most subjects, in contrast to the horizontal montage (see Fig. 3).

**Fig. 5:**
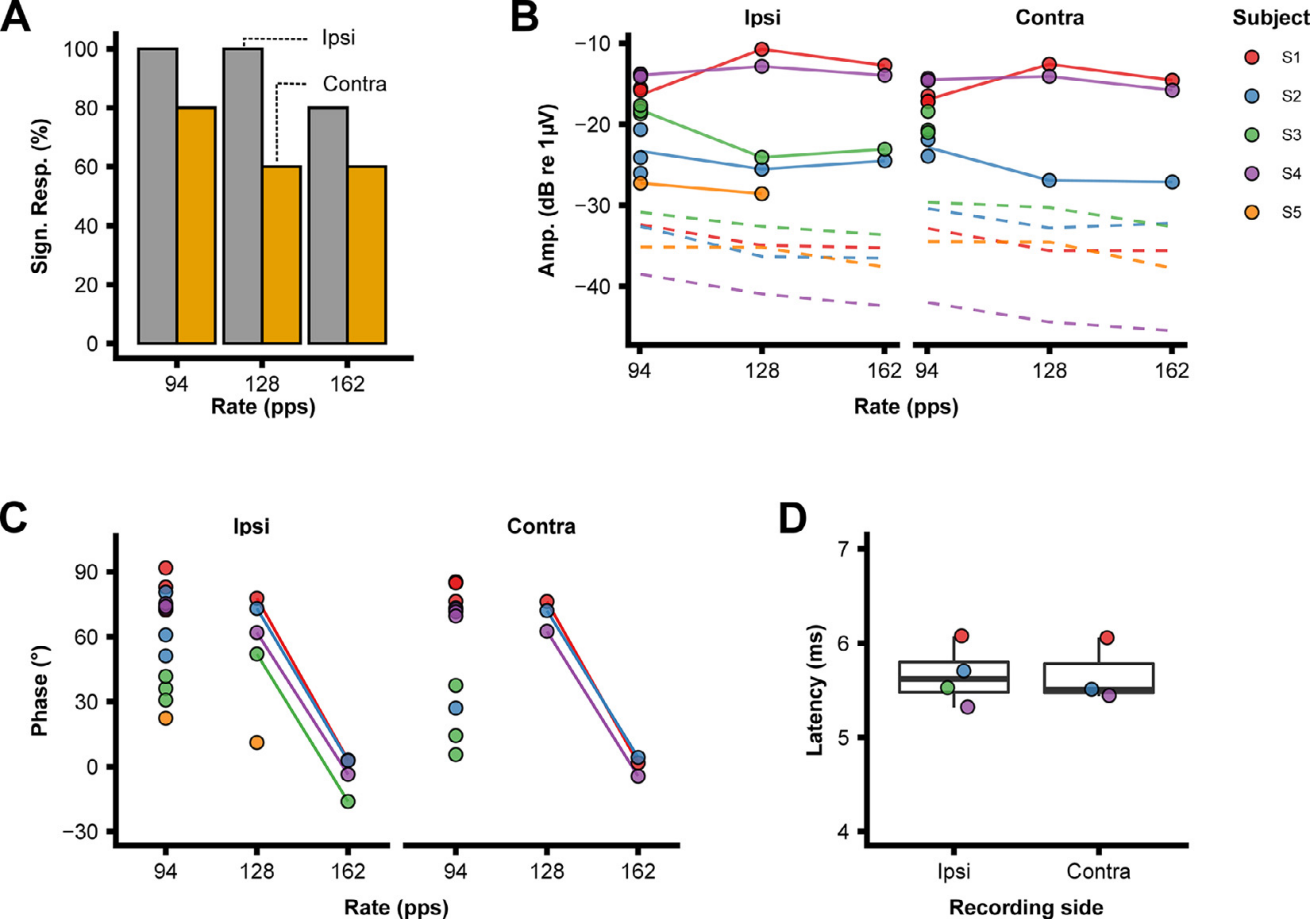
**A)** The number of significant eFFRs as a function of pulse rate each (*n* = 5). **B)** The response amplitude (solid lines) and neural background amplitude (dashed lines) as a function of pulse rate. . The response amplitude line is set to the average of the three measurements at 94 pps, and only significant responses are shown (*p* ≤ < 0.05). **C)** The phase as a function of pulse rate, only significant responses are shown (*p*  <0.05) and the lines represent the phase lag between the 128- and 162-pps eFFRs that are used to calculate the latencies. D) The latencies derived from the phase lag of the ipsi and contralateral positioned recording electrodes. Color coding is the same in **Panel B, C**, and **D**.

Significant eFFRs (*p* <0.05) could be obtained from all subjects to the 94-pps pulse rate. All subjects except S5 had a response to the 162-pps pulse rate (Fig. 5**A**). Response amplitudes across pulse rates and subjects were on average 132 nV (range = 37.3–290 nV) and 136 nV (range = 44.1–235) for eFFRs obtained with respectively the ipsi and contralateral placed recording electrodes. As shown in Fig. 5**B**, subject-specific response patterns were relatively stable across pulse rates. The differences in eFFR phase across pulse rates shows that there is a consistent phase lag between the 128- and 162-pps eFFRs across all subjects, but that this is not the case for the 94-pps eFFR. This is consistent with the results reported by Tichko and Skoe (2017), who found that there is a larger contribution of sources beyond the brainstem in the generation of FFRs < 100 Hz in acoustic hearing. Furthermore, Gransier et al. (2016) reported that the latencies of eASSR, measured in adult CI users, to pulse trains modulated between 80 and 100 Hz had latencies around 15 ms, indicating contributions of generators beyond the brainstem to the scalp recorded eFFR. The slope of the phase lag between the 128- and 162-pps eFFR corresponds to an average latency of 5.7 ms across ipsi- and contralateral recording electrodes (Fig. 5**D**), which is consistent with a predominant midbrain/brainstem source considering that the CI bypasses the basilar membrane.

### 4.3 Auditory change complexes to rate changes

**Fig. 6:**
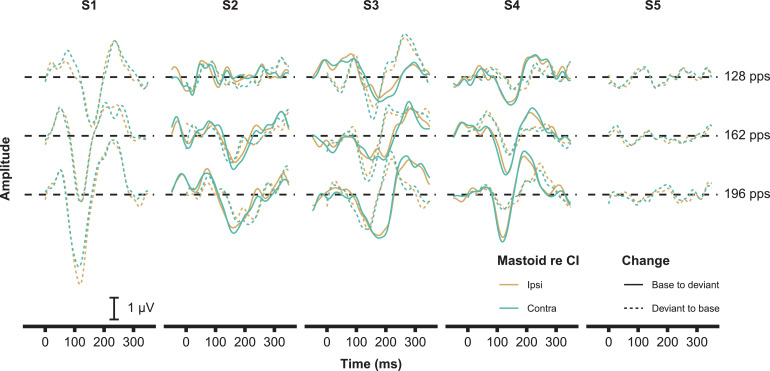
Individual ACC waveforms as a function of rate change. The waveforms have an offset per rate change to aid visibility (horizontal black dashed lines). Subjects S1 and S5 do not have an ACC to the base-to-deviant condition due to compliance issues (see text for details).

**Fig. 7:**
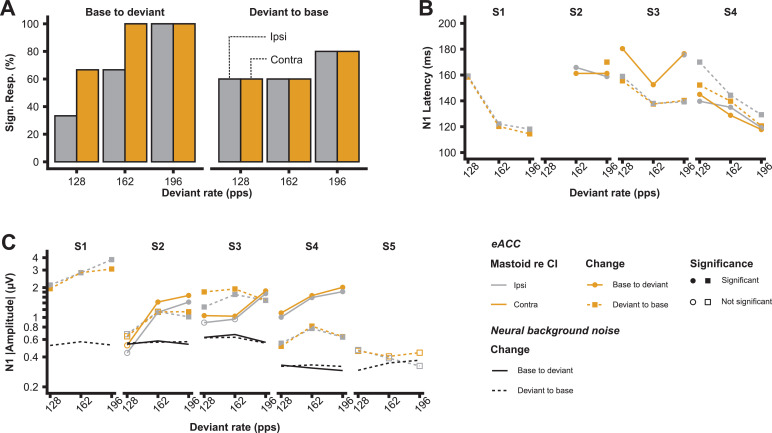
**A)** The number of significant eACCs as a function of deviant pulse rate, note that only 3 subjects were included for the base-to-deviant change. **B)** The response N1 latency as a function of the deviant pulse rate. Only responses that are significantly different from the background noise are shown. **C)** The absolute response amplitude of the N1 peak and neural background noise (mean across electrodes) as a function of the deviant pulse rate. Color coding is the same in A, B, and C.

Fig. 6 shows the individual waveforms to the different rate changes. eACCs could be obtained in four out of five subjects to one or more of the rate changes (see Fig. 7**A** for the percentages). Subject S5 had no measurable eACCs to any of the rate changes. Although the eACC had a different morphology across subjects, the N1 peak was always prominent (Fig. 6). For most subjects there was an increase in N1 amplitude and decrease in N1 latency with the increase of the rate difference between changes for the base-to-deviant rate but not for the deviant-to-base rate (Fig. 7**B & C**). The largest rate change (94–196 pps) resulted in an average N1 amplitude increase of 5.1 dB and a 16 ms decrease in latency of the N1 peak compared to the lowest rate change (94–128 pps).

## Conclusion

This is the first study, to the authors’ knowledge, that demonstrates the feasibility of measuring eFFRs to pulse trains and eACCs to rate changes in adult CI users. Both measures are of potential interest to assess neural rate encoding at the subcortical and cortical levels of the electrically-stimulated auditory pathway, and could be a valuable tool to gain insight in the underlying neural mechanisms that affect rate processing and pitch perception in CI users.

Effective removal of stimulation artifacts ‒inherent to electrical stimulation‒ is challenging, especially when measuring sustained phase-locked electrophysiological responses, like the eFFR. Sustained phase-locked responses have been measured successfully in CI users with eASSR paradigms to modulated pulse trains (Bahmer et al., 2018; Gransier et al., 2020b, 2016; Hofmann and Wouters, 2012). However, the artifact component at the modulation frequency is lower in magnitude than at the carrier rate (Gransier et al., 2020b), with the result that shorter blanking lengths are needed to obtain artifact free eASSRs compared to artifact free eFFRs. Although differences in artifact duration exists across CI users, we found that a 3.5-ms blanking length was effective to obtain artifact-free eFFRs when using the hyper-rate EEG system in all participants. In contrast, the blanking method can effectively be applied for the recording of artifact-free eASSRs to modulated pulse trains with pulse rates of 500 to 900 pps and stimulated in monopolar mode (Bahmer et al., 2018; Gransier et al., 2020b, 2016). Furthermore, we found that the blanking length required to remove the stimulation artifacts adversely affects the neural background noise and the eFFR evoked with the 196-pps pulse train. This limits the effectiveness and applicability of the blanking method for artifact removal when using pulse rates >162 pps and stimulating in monopolar mode. These effects were also observed in the simulations. For the assessment of eFFRs to higher pulse rates artifact removal techniques that do not affect the neural response [e.g. template subtraction (Deprez et al., 2017a)] need to be developed and evaluated. On the other hand, different stimulation modes could also be considered or alternating polarity. The latter can, however, be challenging since loudness differences can exist between polarities. Stimulation-wise, bipolar mode, although less used clinically, has a smaller stimulation artifact and requires shorter blanking durations (Hofmann and Wouters, 2010) than monopolar stimulation. Nevertheless, the pulse rates ≤ 162 pps that can be used to record artifact-free eFFRs with monopolar stimulation are well within the F0 range of speech stimuli normally used to evoke FFRs acoustically (Krizman and Kraus, 2019). Furthermore, the latency of eFFRs evoked with rates of 128 and 162 pps were around 5 ms, which is consistent with a predominant brainstem generator (King et al., 2016). The eFFR can therefore be an interesting noninvasive tool to probe the rate encoding ability of the brainstem in CI users.

We were able to measure eACCs to rate changes in four out of five subjects. Each of these subjects had an eACC to one or more rate changes. Although a difference in waveforms morphology was present across subjects, the N1 responses ‒ which has its main generators located within the auditory cortex ‒ was always present in case of statistically significant eACCs. An overall increase in N1 amplitude and decrease in N1 latency was observed when the difference between the two pulse rates changed from 36% to 108%. Similar increases in ACC strength and decreasing latencies have been reported in other change paradigms e.g., electrode discrimination (Mathew et al., 2018; R. 2017) and modulation encoding (Gransier et al., 2020a; Han and Dimitrijevic, 2020; Undurraga et al., 2020) in CI users. Given the cortical nature of the eACC it can potentially be used to probe the sensitivity of the cortex to rate changes. One of the advantages of the eACC is that it can very easily be recorded and different approaches can be used to remove the stimulation artifact from the recording (Mc Laughlin et al., 2013). One has to consider, however, when using eACCs to assess rate encoding in CI users, that the absence of an eACC is not direct evidence for a reduced sensitivity of the auditory cortex, but that it could also be a result of poorer rate encoding at the peripheral and subcortical levels of the auditory pathway. By combining both the eFFR and the eACC as used here, one is able to gain insight in the rate encoding abilities of both the brainstem and auditory cortex. The combination of these measures could potentially provide insight in neural mechanisms that contribute to rate and pitch processing in CI users.

## Appendix Supplementary materials

Image, application 1

## Footnotes

1 Cochlear Ltd uses Current Levels to set the current delivered by the implant. The current can be increased in 255 integer steps. Ten current levels correspond to approximately 1.8 dB difference in current.
