# Supplementary material for: Frequency following responses and rate change complexes in cochlear implant users
Source: Hear Res. 2021 May;404:108200. doi: 10.1016/j.heares.2021.108200 (PMC8052190; doi:10.1016/j.heares.2021.108200)
Supplement: Supplementary file 1 [file mmc1.docx]

**
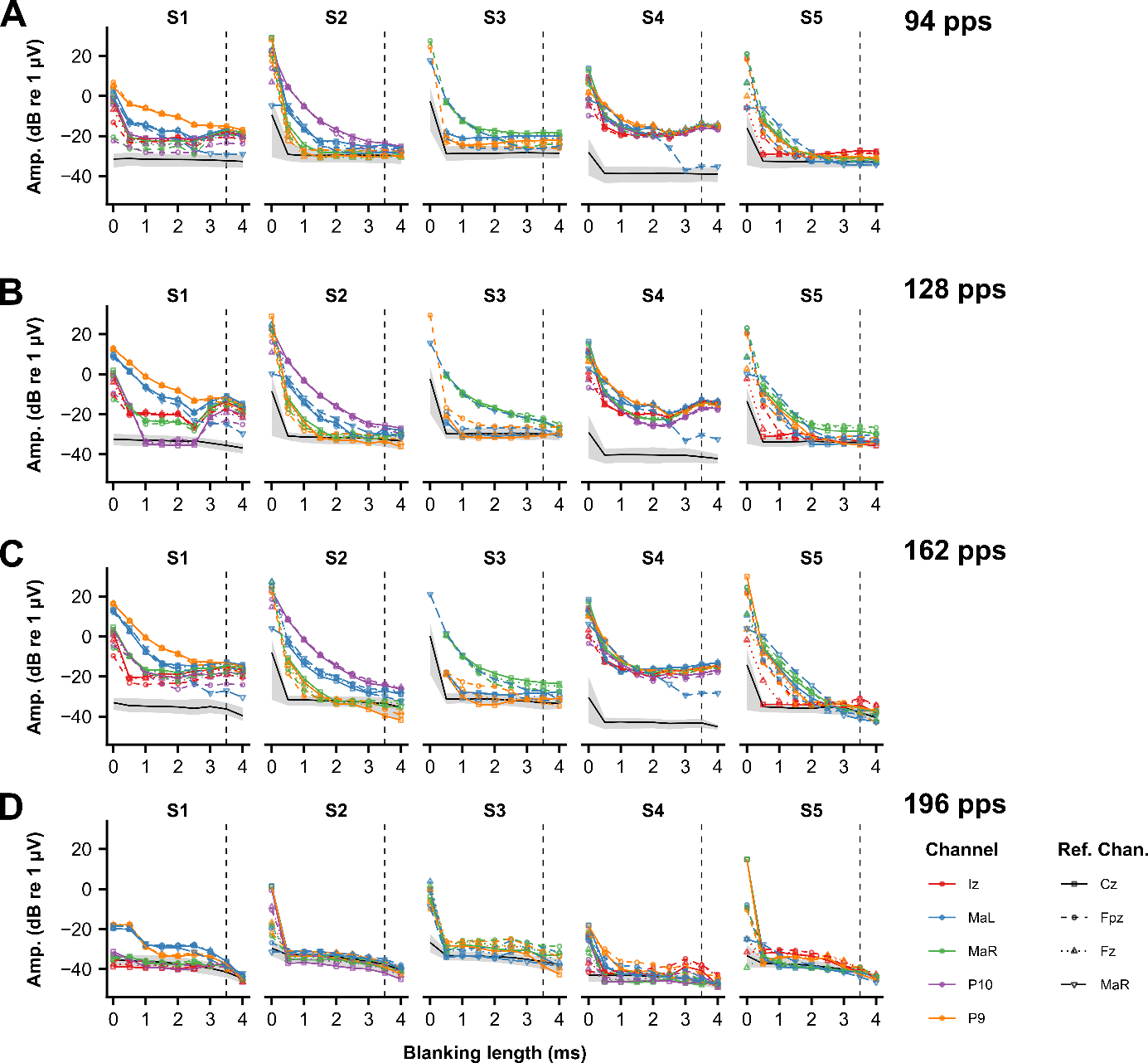
**

**Supplementary Figure 1:** Artifact removal assessment showing the response amplitude at the stimulation rate (**A**: 94, **B**: 128, **C**: 164, **D**: 196) as a function of the blanking length for each individual subject. Colors show the different non-inverting channels and the different symbols and line types show the different reference channels (Ref. Chan.). The solid black line and the shaded area shows the average, and range of noise levels across all recording-electrode configurations as a function the blanking length, respectively. The dashed vertical line shows the blanking length used in the analysis of the eFFR. Response amplitudes that were lower than the noise level for a specific combination (i.e. recording-electrode configuration and blanking length) were set to the noise level of that specific combination for illustrative purposes.


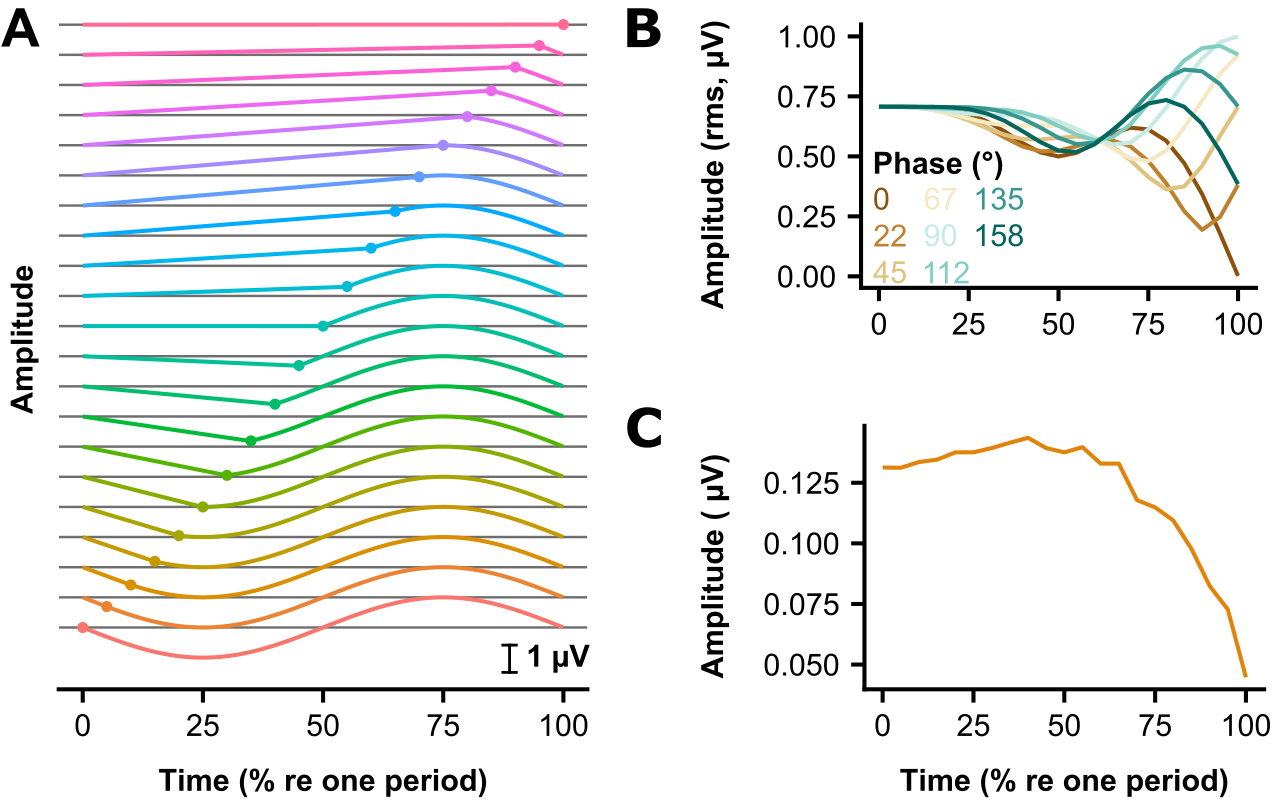


**Supplementary Figure 2: A)** Simulations of the effect of blanking on a sinusoidal response waveform with a starting phase of 0 degrees. The different waveforms show the different blanking lengths, and the blanking length is shown by the marker on each waveform. **B)** The RMS value as a function of the blanking length for sinusoids with different starting phases. **C).**  The mean noise level across epochs (n = 300) at the frequency bin that corresponds to the pulse rate (100 pps) as a function of blanking length.

**Supplementary Table I:** The number of epochs used per subject and per condition for the analysis of the electrophysiological responses after artifact rejection.

| **Subject** | **Deviant Rate** | **Epochs** |
| --- | --- | --- |
| S1 | 128 | 386 |
| S1 | 162 | 387 |
| S1 | 196 | 387 |
| S2 | 128 | 384 |
| S2 | 162 | 385 |
| S2 | 196 | 384 |
| S3 | 128 | 288 |
| S3 | 162 | 288 |
| S3 | 196 | 340 |
| S4 | 128 | 388 |
| S4 | 162 | 388 |
| S4 | 196 | 385 |
| S5 | 128 | 384 |
| S5 | 162 | 480 |
| S5 | 196 | 480 |
